# Subcutaneous Facial Mass as a Rare Presentation of Follicular Lymphoma: A Case Report

**DOI:** 10.7759/cureus.79770

**Published:** 2025-02-27

**Authors:** Geena Jung, Christopher V Lisi, Shanmugappiriya Sivarajah, Svetoslav Bardarov, Wahib Zafar

**Affiliations:** 1 College of Medicine, Albert Einstein College of Medicine, Bronx, USA; 2 Otolaryngology-Head and Neck Surgery, Richmond University Medical Center, Staten Island, USA; 3 Pathology and Laboratory Medicine, Richmond University Medical Center, Staten Island, USA; 4 Hematology and Oncology, Richmond University Medical Center, Staten Island, USA

**Keywords:** biopsy, clinical management, facial mass, fine needle aspiration, follicular lymphoma

## Abstract

Follicular lymphoma is a common form of non-Hodgkin lymphoma. Extranodal involvement, especially in soft tissue, is rare. Here, we present a case of a 68-year-old female patient who presented with a recurrent swollen cheek, which was initially misdiagnosed as seasonal allergies. Imaging revealed a soft tissue mass, but two fine needle aspiration (FNA) biopsies were negative for malignancy. Clinical suspicion remained high and surgical excision was performed, revealing lymphoid proliferation consistent with follicular large B-cell lymphoma. Our case underscores the diagnostic challenges of soft tissue lymphomas and limitations of FNAs in ruling out malignancy. Furthermore, it highlights the necessity of maintaining clinical suspicion for lymphoma in cases of unresponsive facial masses.

## Introduction

Follicular lymphoma (FL) is a common indolent subtype of non-Hodgkin lymphoma (NHL) and accounts for between 20 and 35% of all cases of NHL [1-3]. The median age of diagnosis is between 60 and 65 years; the neoplasm is uncommon in children [4,5]. A majority of patients with FL have the chromosomal translocation t(14,18)(q32;q21), which ultimately results in an over-proliferation of B-cells [6].

Typically, patients with FL have diffuse waxing and waning lymphadenopathy [7]. Approximately 70% of patients will also have bone marrow involvement [8]. In about 20% of cases, patients may present with B symptoms such as fever, night sweats, and weight loss [9,10]. Extranodal involvement in patients with FL is not common. 

Here, we report a rare and complex case of follicular large B-cell lymphoma (FLBCL) in the form of a right subcutaneous facial mass. We describe the presentation, diagnosis, and management of the lesion.

## Case presentation

The patient was a 68-year-old woman who presented with a slowly enlarging, 3x1 cm fixed mass in the right cheek, which had originally appeared two years ago. She had a history of stage 1 breast cancer treated with lumpectomy followed by chemotherapy and radiation, skin cancer on the back treated with surgical resection, osteoarthritis, and psoriasis. When the cheek swelling first began, it was attributed to seasonal allergies, leading to the administration of oral steroids. The mass initially resolved with steroid treatment but recurred. The patient denied any skin lesions or rash over the lesion, night sweats, fever or chills, recurrent illnesses, cough, or jaw pain.

**Figure 1:**
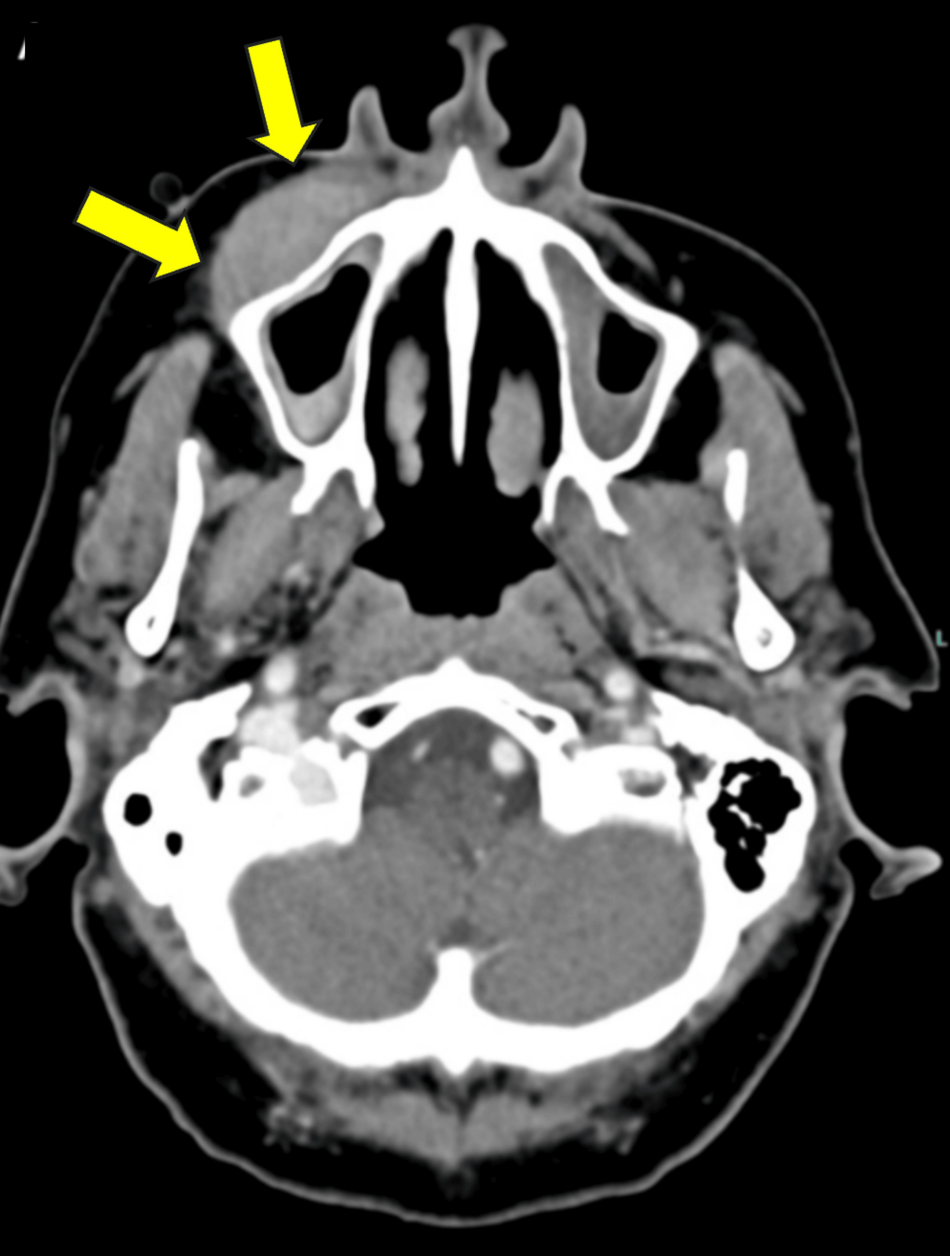
Coronal computed tomography scan with contrast of the right facial mass prior to surgical treatment. There is a 3.3 by 1.2 cm soft tissue mass abutting the anterior wall of the right maxillary sinus. No underlying osseous destruction is seen. The soft tissue of the nasopharynx, orbits, and brain is unremarkable.

**Figure 2:**
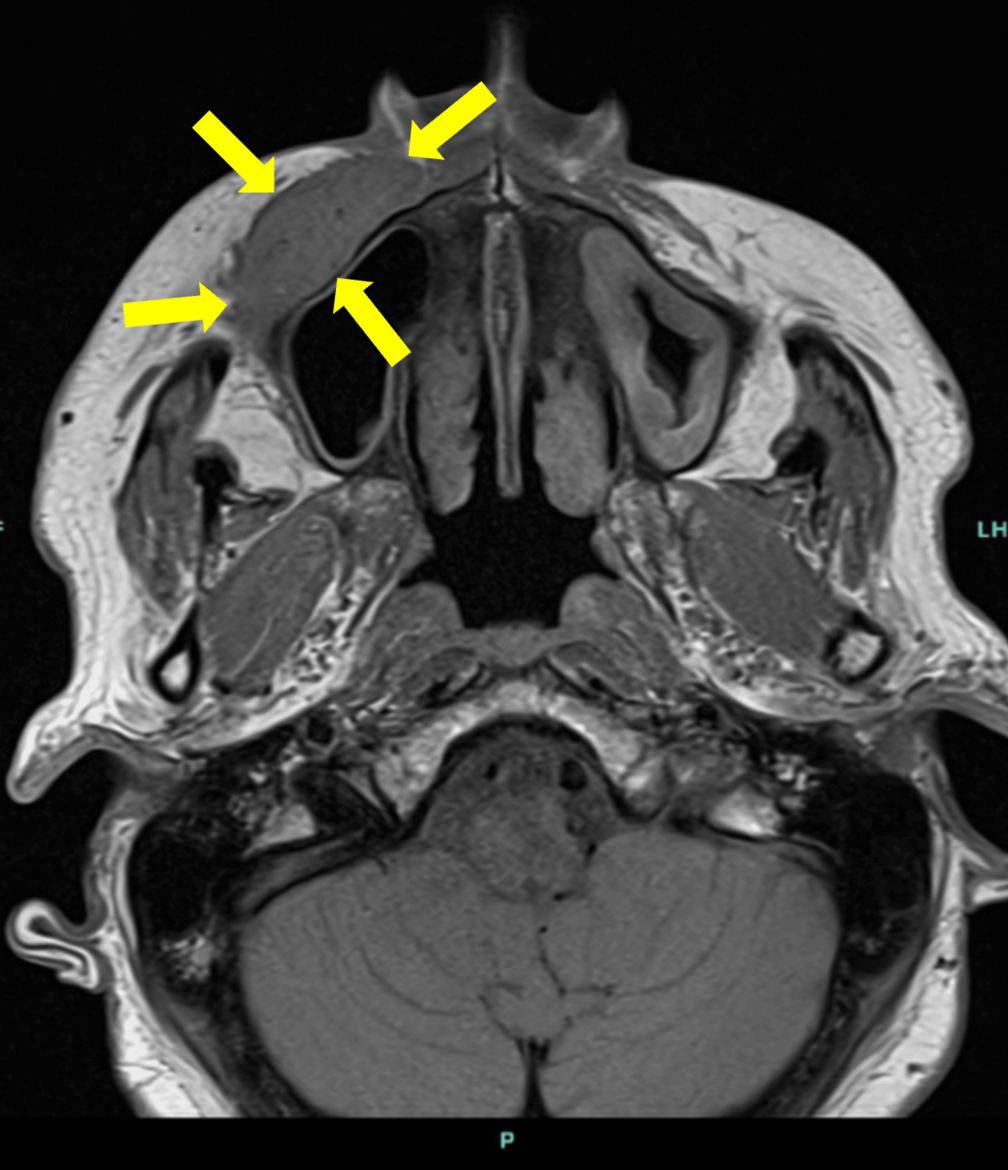
Coronal T1-weighted magnetic resonance imaging of the right facial mass (arrows) prior to surgical treatment. There is a well-delineated soft tissue mass within the right cheek soft tissue measuring approximately 3.5 x 1.5 cm in maximal length and depth.

A CT scan showed 3.3x1.2 cm soft tissue density, with no underlying osseous destruction or definite adjacent fat stranding (Figure 1). Further evaluation with an MRI scan showed a well-delineated soft tissue mass within the right cheek soft tissue overlying the anterior wall of the right maxillary antrum, measuring approximately 3.5x1.5x3.5 cm (Figure 2). Two fine needle aspiration (FNA) biopsies were performed, and both showed no evidence of B- or T-cell lymphoproliferative disorder. The B cells expressed the pan B-cell markers CD19 and CD20 and lacked CD5 and CD10. The B cells were polyclonal with regard to light chain determinants. The T-cell population expressed CD5 in a non-aberrant fashion. There was no evidence for monoclonal B-cells or atypical T-cell populations.

Due to the persistence of the mass and the uncertainty of its origin, the mass was surgically excised. The mass was not enclosed by a capsule and extended to the masseter with irregular borders. The buccal branch of the facial nerve and two branches of the trigeminal nerve were sacrificed to achieve adequate margin. Frozen sections showed clear margins. 

**Figure 3:**
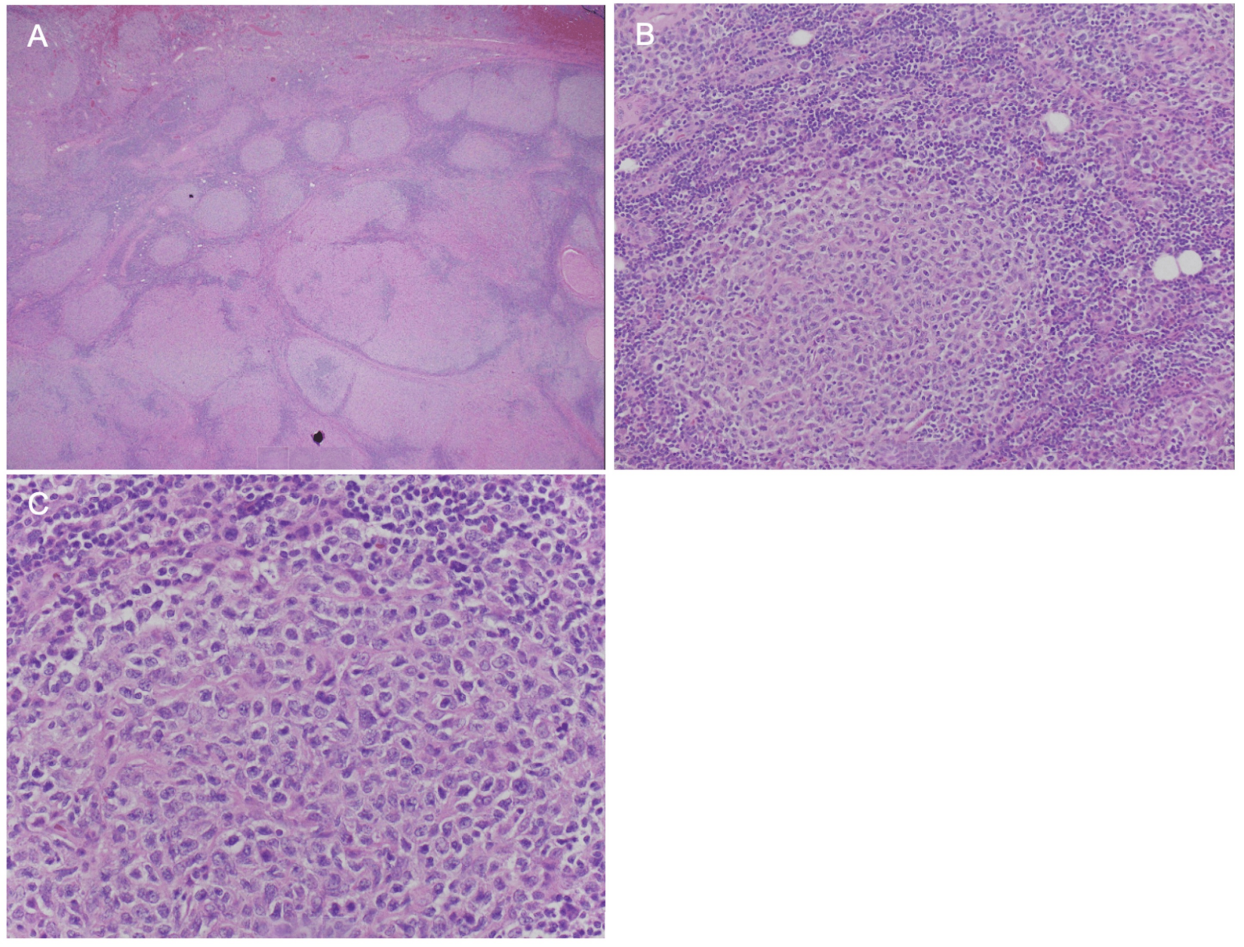
Histologic staining of the resected mass. Hematoxylin and Eosin (H&E) staining in 2x of follicular large B-cell lymphoma (A). H&E staining in 20x of follicular large B-cell lymphoma demonstrating expanded germinal centers (B). H&E staining in 40x of follicular large B-cell lymphoma showing germinal centers with an increased number of centroblastic-type cells (C).

Histopathological examination of the sections revealed an expanded nodular lymphoid proliferation within fibroadipose tissue, lacking a definite capsule or adnexal structures (Figure 3). The lymphoid proliferation displayed a nodular pattern with numerous centroblastic-type cells. Immunohistochemistry results showed that the lymphoma cells were positive for CD20, CD10, BCL-2, and BCL-6 and negative for BCL-1 and CD5. The proliferation index, measured with Ki-67 immunostaining, ranged from 10 to 20%.

The differential diagnosis includes primary cutaneous follicle center lymphoma and FLBCL. The expression of BCL-2 supports a diagnosis of FLBCL. Additionally, fluorescent in situ hybridization detected the t(14;18) translocation in 40% of the tumor cells, further supporting the diagnosis of FLBCL, stage 1. 

Subsequent PET scans were negative for distal disease. The patient was seen by hematology/oncology, who performed a bone marrow biopsy which also came back negative. Currently, the patient is being seen by radiation oncology for radiation treatment. She has right-sided facial numbness and facial weakness, as well as tearing of the eyes with right eyelid droop, all of which has been improving.

## Discussion

NHLs typically present with diffuse lymphadenopathy in the absence of systemic symptoms. Occasionally, they can involve extranodal sites, though this is not common. Lymphomas involving soft tissue, including adipose tissue, are extremely rare events and have been reported to occur in approximately 0.1% of all lymphomas [11,12]. Reports from most cases of the literature found diffuse large B cell lymphoma to be the most common histologic subtype of soft tissue NHLs [12]. In cases where soft tissue is involved, it is typically due to extranodal involvement or direct extension from affected lymph nodes [13].

Here, we report a rare case of FLBCL with an initial presentation of a subcutaneous facial mass. Given the rarity of soft tissue lymphomas, the mass was initially thought to be attributed to allergies, and oral steroids were prescribed. The mass recurred despite transient improvement with steroids, and CT and MRI confirmed the presence of a soft tissue mass within the right cheek overlying the maxillary antrum. Despite two negative FNAs, clinical suspicion for malignancy was still high. As such, prompt action was taken by an interdisciplinary team consisting of ENT, pathology, medical oncology, and radiation oncology to develop a plan of action. Discussions with the team led to a final decision on surgical resection and radiation treatment. Ultimately, the mass was found to be consistent with FL.

Our case highlights that even though FL of the soft tissue of the face is rare, it should be on the differential for a patient with a subcutaneous facial mass. This is especially true in patients with a history of cancer and chemotherapy or radiotherapy. It also underscores the limitations of FNAs; an FNA that does not show evidence of lymphoma does not rule out malignancy, as seen in our patient. Clinical suspicion for malignancy should remain high, especially when there are other indications of a potential underlying neoplastic process. In our case, the persistence of the mass despite treatment with steroids and supportive treatment heightened clinical suspicion of an underlying malignancy. Given these concerns, an excisional biopsy was necessary in order to obtain a larger tissue sample for histopathological diagnosis.

Moving forward, the patient will undergo radiation therapy and follow-up protocols as per National Comprehensive Cancer Network (NCCN) guidelines [14]. Updated clinical NCCN guidelines dictate that if there is a complete or partial response to radiation therapy, clinical follow-up includes the following: history and physical (H&P) and labs every 3-6 months for five years, then once every year or as clinically indicated. The patient will also undergo surveillance imaging up to two years following treatment completion with CT scans of the chest/abdomen/pelvis every six months and an annual scan after two years.

## Conclusions

FL of the soft tissue is an extremely rare event. Here, we present a case of FLBCL in the form of a subcutaneous right facial mass. Despite two FNAs showing no evidence of malignancy, clinical suspicion remained high and the mass was excised. From our case, we highlight that lymphomas should be on the differential for patients with facial soft tissue masses, especially for lesions that do not respond to conservative treatment and/or recur. Moreover, our case demonstrates that FNAs alone cannot be used to rule out malignancy.
